# Urea Transporter UT-B Deletion Induces DNA Damage and Apoptosis in Mouse Bladder Urothelium

**DOI:** 10.1371/journal.pone.0076952

**Published:** 2013-10-21

**Authors:** Zixun Dong, Jianhua Ran, Hong Zhou, Jihui Chen, Tianluo Lei, Weiling Wang, Yi Sun, Guiting Lin, Lise Bankir, Baoxue Yang

**Affiliations:** 1 State Key Laboratory of Natural and Biomimetic Drugs, Key Laboratory of Molecular Cardiovascular Sciences, Ministry of Education, and Department of Pharmacology, School of Basic Medical Sciences, Peking University, Beijing, China; 2 Department of Anatomy, Neuroscience Research Center, Basic Medical College, Chongqing Medical University, Chongqing, China; 3 Department of Urology, University of California San Francisco, San Francisco, California, United States of America; 4 INSERM Unit 872, Centre de Recherche des Cordeliers, Paris, France; Emory University, United States of America

## Abstract

**Background:**

Previous studies found that urea transporter UT-B is abundantly expressed in bladder urothelium. However, the dynamic role of UT-B in bladder urothelial cells remains unclear. The objective of this study is to evaluate the physiological roles of UT-B in bladder urothelium using UT-B knockout mouse model and T24 cell line.

**Methodology/Principal Findings:**

Urea and NO measurement, mRNA expression micro-array analysis, light and transmission electron microscopy, apoptosis assays, DNA damage and repair determination, and intracellular signaling examination were performed in UT-B null bladders *vs* wild-type bladders and *in vitro* T24 epithelial cells. UT-B was highly expressed in mouse bladder urothelium. The genes, Dcaf11, MCM2-4, Uch-L1, Bnip3 and 45 S pre rRNA, related to DNA damage and apoptosis were significantly regulated in UT-B null urothelium. DNA damage and apoptosis highly occurred in UT-B null urothelium. Urea and NO levels were significantly higher in UT-B null urothelium than that in wild-type, which may affect L-arginine metabolism and the intracellular signals related to DNA damage and apoptosis. These findings were consistent with the *in vitro* study in T24 cells that, after urea loading, exhibited cell cycle delay and apoptosis.

**Conclusions/Significance:**

UT-B may play an important role in protecting bladder urothelium by balancing intracellular urea concentration. Disruption of UT-B function induces DNA damage and apoptosis in bladder, which can result in bladder disorders.

## Introduction

Urea transporters (UT) belong to a family of membrane proteins that selectively transport urea driven by urea gradient across the membrane. In mammals, at least seven urea transporters, UT-A1 to UT-A6 and UT-B, have been characterized [1]. UT-B is expressed in multiple tissues including erythrocyte, kidney, brain, heart, colon, spleen, testis, ureter, bladder, etc [2], and expressed in bladder at the highest level among all tissues. Previous studies showed a “urea-selective” urinary concentrating defect in UT-B null mice [3], [4]. UT-B deletion also causes urea accumulation in testis and early maturation of the male reproductive system [5]. Additionally, cardiac conduction defects were found in aged UT-B null mice with prolonged P–R interval and action potential duration [6]. Depression-like behavior was also found in UT-B null mice. Furthermore, UT-B deletion induces alteration in nitric oxide synthase (NOS)/nitric oxide (NO) system [7]. However, whether UT-B deficiency influences the function of urinary bladder still remains unknown.

Bladder has long been regarded as a transit and storage organ for urine in mammals. Normally, bladder urothelial cells are exposed to an inclement environment for a long time, such as high osmolality and high urea concentration. Urea is highly concentrated in urine up to more than a thousand of mmol/l that represents about 45% of total urinary solutes. It is difficult to understand the role of urea transporter UT-B in bladder urothelial cells, but the presence of UT-B in these cells may suggest urea is transported across bladder urothelium [8]. Without UT-B in the basal membrane of bladder urothelium, we suggest that urea might accumulate in urothelial cells at an abnormally high concentration causing a disturbance in normal bladder function. Interestingly, it has been found that UT-B gene mutations were related to bladder carcinogenesis in human [9], [10].

To determine the functional consequence of UT-B deficiency on urothelial cells, we examined differential gene expressions and phenotypes in UT-B null and wild-type bladder urothelium. It was found that DNA damage and apoptosis were significantly increased and that the expression of some related genes was greatly altered in UT-B null urothelial cells. Deletion of UT-B induced urea accumulation, an up-regulated expression of iNOS and a down-regulation of arginase I expression in urothelial cells. These results suggest that UT-B deficiency is responsible for a marked elevation of urea concentration in bladder urothelial cells. This intracellular urea accumulation creates an imbalance between the arginine-ornithine-polyamine pathway and the arginine-citrulline-NO pathway. thus rendering these cells more vulnerable to DNA damage and apoptosis.

## Materials and Methods

### Animals

UT-B knockout mice were generated by targeted gene disruption as previously reported [3]. UT-B null mice did not express detectable UT-B protein in any organ. The mice used in this study had a C57BL/6J genetic background. Mice were housed at constant room temperature (23±1°C) and relative humidity (50%) under a regular light/dark schedule (light on from 7∶00 A.M. to 7∶00 P.M.) with freely accessible food and water before experiments. Protocols were approved by Peking University Health Center, Committee on Animal Research.

### Cell Culture

T24 cells (human urinary bladder epithelial cell line, ATCC^#^: HTB-4™) were cultured in RPMI medium 1640 (GIBCO) containing 10% fetal bovine serum, 1% penicillin/streptomycin, and incubated at 37°C in 5% CO_2_ atmosphere. The cells were seeded at 2×10^4^ cells per 60-mm plate.

### Sample Preparation

Mice, 8 weeks old, were deeply anesthetized with pentobarbital (85 mg/kg, i.p.). Their urinary bladder was carefully removed and fixed overnight in 4% paraformaldehyde for immunostaining and TUNEL assay. The specimens were transferred to a solution containing 30% sucrose in 0.1 M phosphate buffer, pH 7.4. For Western blot, bladder urothelial cells were collected directly, homogenized in 1X RIPA lysis buffer (Thermal Scientific, 89901) containing 1X protease inhibitor cocktail (Roche, 11873580001).

### Microarray Analysis

Total RNA from the UT-B null and wild-type mouse bladder urothelium was isolated with the RNAeasy Isolation Kit (Qiagen). All RNA used in this experiment were of high quality as indicated by a ratio of 2∶1 for 28S/18S rRNA and a ratio of >1.9 for OD_260_/OD_280_. One microgram of UT-B null mouse urothelial RNA, side by side with 1 µg of wild-type mouse urothelial reference RNA, were linearly amplified through two rounds of modified *in vitro* transcription that had been testified by the technique control. The amplified messenger RNA and coupling were performed. The labeled urothelial and reference complementary DNA (cRNA) probes were combined and hybridized to a mouse oligo microarray slide at 48°C for 12–16 hr. The microarray slides were scanned with Axonimager 4000B and GenePixPro 6 software (Axon Instruments, Union City, CA, USA). GenePix median of ratios was subjected to linear normalization in NOMAD (http://derisilab.ucsf.edu); the normalized data were then analyzed with Cluster 3.0 (http://bonsai.ims.utokyo.ac.jp/~mdehoon/software/cluster/). The resulting cluster data were imported into the Significance Analysis of Microarrays (SAM) software package (http://www-stat.stanford.edu/~tibs/SAM/). Option Delta was chosen to limit the output gene list so that less than 1% of predicted false positives would be included.

### Cell Viability Assay

T24 cells were harvested using 0.25% trypsin. Cells were suspended (20,000 cells/ml, 0.1 ml/well) in DMEM containing 10% FBS, plated onto gelatinized 96-well culture plates, and incubated at 37°C, 5% CO_2_ for 24 hours. The media were replaced with 0.1 ml of DMEM without FBS and incubated for 24 hours with urea at different concentrations (5, 37.5, 75, 150 or 300 mmol/l). Cell viability was assayed according to the manufacturer’s instructions from CCK-8 Kit (Dojindo, Japan).

### Transmission Electron Microscope

Mice were deeply anesthetized with pentobarbital (85 mg/kg, i.p.) and perfused with 4% glutaraldehyde via the heart. One cubic millimeter tissue blocks were isolated from the bladder. The dehydrated samples were embedded with TEM of Epoxy Resin Embedded Tissue Kit (Genmed Gene Pharmaceutical Co., Ltd, Shanghai, China) and polymerization. Semithin sections were cut with a LKB III ultratome, stained with 1% Toluidine Blue, and inspected to determine orientation. Ultrathin sections were cut with a Leica Ultracut Ultramicrotome (German), stained with uranyl acetate and lead citrate, and examined at 80 kV with HITACHI-7500 electron microscope (Hitachi Instruments Ltd., Tokyo, Japan).

### Urea Measurement

Urea concentration in bladder urothelium was measured as described by Spetor et al [8] with some modification. Mice were anesthetized with pentobarbital (85 mg/kg, i.p.) followed by the removal of the bladder. To remove residual urine from the luminal surface, bladders were opened longitudinally, quickly rinsed with NaCl solution (150 mmol/l), and blotted three times on absorbant paper. The bladder urothelial samples were collected, weighed and homogenized in 100 µl of distilled water and centrifuged at 12,000×g for 15 min at 4°C. Urea concentration from the supernatant was measured using quantitative colorimetric Urea Determination Kit (QuantiChrom™ Urea Assay Kit-DIUR-500). Results were expressed relative to fresh tissue weight.

### NO Content Measurement

For the measurement of NO in urothelium, the bladder urothelial samples were prepared as mentioned above for urea. NO concentration was measured in the supernatant and expressed relative to the tissue weight in the same way as for urea. For the measurement of NO released by T24 cells, cells were cultured in 24-well plates. After 3 hours of incubation with or without urea, the suspension was collected for NO content measurement [11]. NO concentrations were determined by NO Assay Kit (Jiancheng Bioengineering Co., Nanjing, China).

### Immunofluorescence

Bladder sections were cut at 6 µm on a cryostat. The sections were blocked with 1% (w/v) bovine serum albumin, 0.1% Triton X-100, and 0.05% Tween-20 overnight at 4°C to avoid unspecific staining. Then, the sections were incubated with rabbit polyclonal antibody, anti-UT-B (UT-B antibody is a kindly gift from Dr. Trinh-Trang-Tan, INSERM, Paris, France). The secondary antibody (Cy3-goat-anti-rabbit, 1∶200) was added in the dark and incubated for 1 hour. The images were captured by Leica fluorescence microscope (Germany).

### TUNEL Staining

Bladder sections were cut at 6 µm on a cryostat and stained with TUNEL Staining Kit (Beyotime) following the manufacturer’s instruction. The images were captured by Leica fluorescence microscope (Germany). Positive signals were quantified in the stained sections by visual field counting (40X). The numbers of positive signals in five sections per mouse from six mice were averaged.

### Western Blot Analysis

Total protein was assayed using Bradford method and size separated by sodium dodecyl sulfate–polyacrylamide gel electrophoresis. Proteins were blotted to polyvinylidene difluoride membranes (Amersham Biosciences). Blots were incubated with polyclonal antibodies against UT-B, ubiquitin C-terminal hydrolase-L1 (Uch-L1) (Bioworld), caspase-3 (Cell Signaling Transduction), Bcl-2 (Santa Cruz), Bax (Santa Cruz), rH2AX (Epitomics), p53 (Sigma), p-p53 (Cell Signaling Transduction), MCM2 (Epitomics), ERK2 (Santa Cruz), p-ERK1/2 (Santa Cruz), iNOS (Cell Signaling Transduction), arginase I (Abcam), arginase II (Santa Cruz) or p-ATM (Ser1981) (Rockland). Goat anti-rabbit IgG (Abcam) and goat anti-mouse IgG (Santa Cruz) were added, respectively, and the blots were developed with ECL Plus Kit (Amersham Biosciences). Relative protein expression levels were quantified by optical density analysis.

### Flow Cytometric Analysis of Cell Cycle and Apoptosis

Cells (2×10^6^) were harvested and washed in PBS. For cell cycle analysis, cells were fixed in 70% ethanol, and stained with propidium iodide [40 µg/ml propidium iodide (PI), 100 µg/ml RNaseA in calcium- and magnesium-free PBS]. For apoptosis analysis, cells were resuspended in binding buffer (10 mmol/l HEPES/NaOH, pH 7.4, 140 mmol/l NaCl, 2.5 mmol/l CaCl_2_). 10 µl of Annexin V-FITC was added to each sample and the cells were incubated in the dark for 30 minutes, then resuspended in 300 µl of binding buffer, and 5 µl of PI were added before flow cytometric analysis. The cells (10,000 independent events) were analyzed for their DNA content with a fluorescence-activated cell sorter (FACS caliber, Becton Dickinson).

### Statistical Analyses

Statistical analysis was performed using one-way ANOVA. *p*<0.05 was considered statistically significant.

## Results

### Expression Localization of UT-B in Mouse Bladder

**Figure 1:**
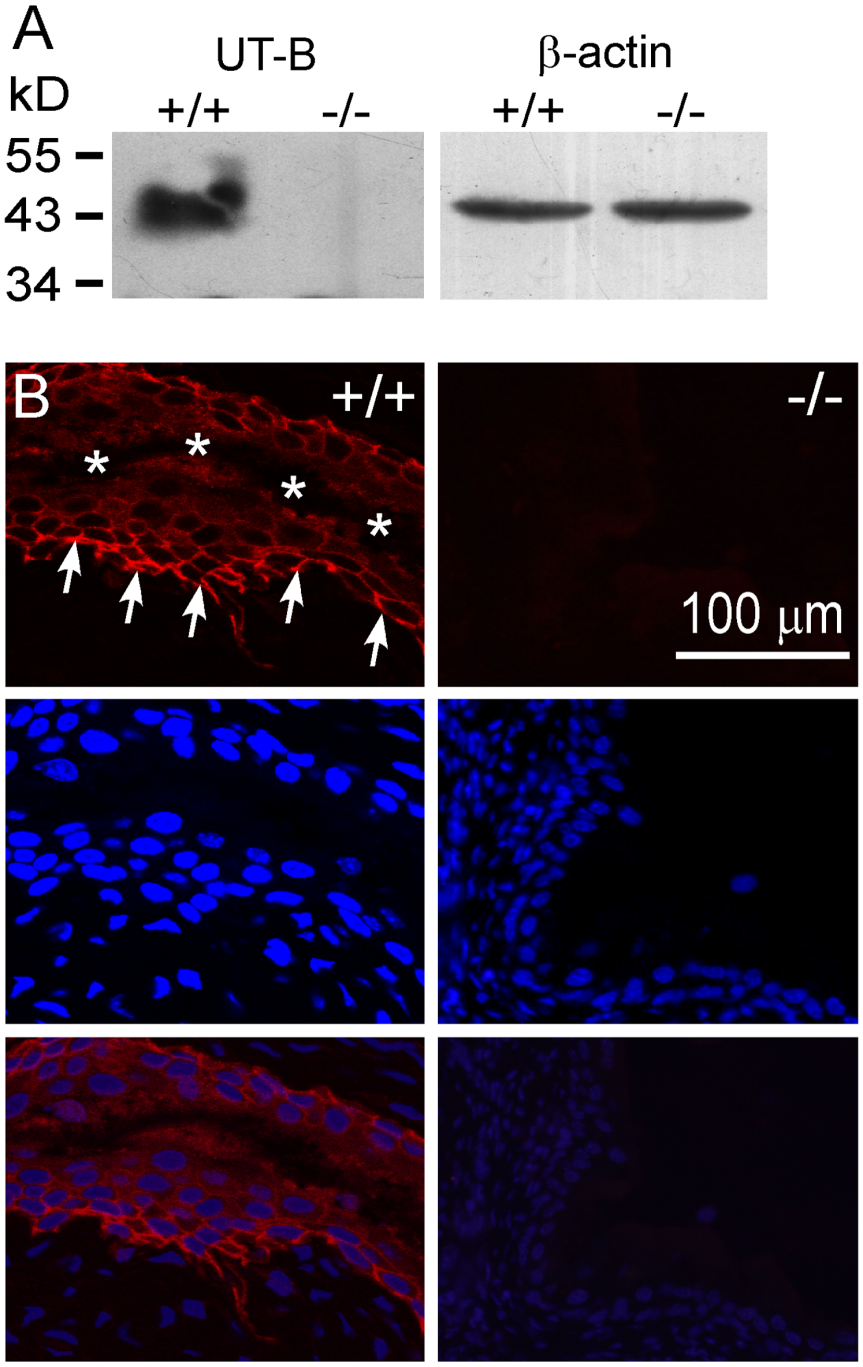
UT-B expression and localization in mouse bladder. **A.** Western blot analysis with UT-B antibody in wild-type (+/+) and UT-B null (−/−) bladder urothelium. **B.** (left) Immunofluorescence showed UT-B (Cy3-labeled) localized in wild-type bladder urothelial cell membrane with cell nuclei stained (blue, DAPI stain). (right) No UT-B was expressed in UT-B null bladder urothelium. Scale bar, 100 µm. Arrows, basal layer of urothelium. *, collapsed luminal space of the bladder.

Western blot analysis probed with UT-B antibody showed a 41∼54-kDa protein expressed in bladder urothelium from wild-type mouse but not from UT-B knockout mouse (Fig. 1A). Immunofluorescence assay showed that UT-B was only expressed in urothelium, and not in the laminae propria or muscular layer as described previously [12] (Fig. 1B).

### Differential Gene Expression in UT-B Null Bladder Urothelium

**Figure 2:**
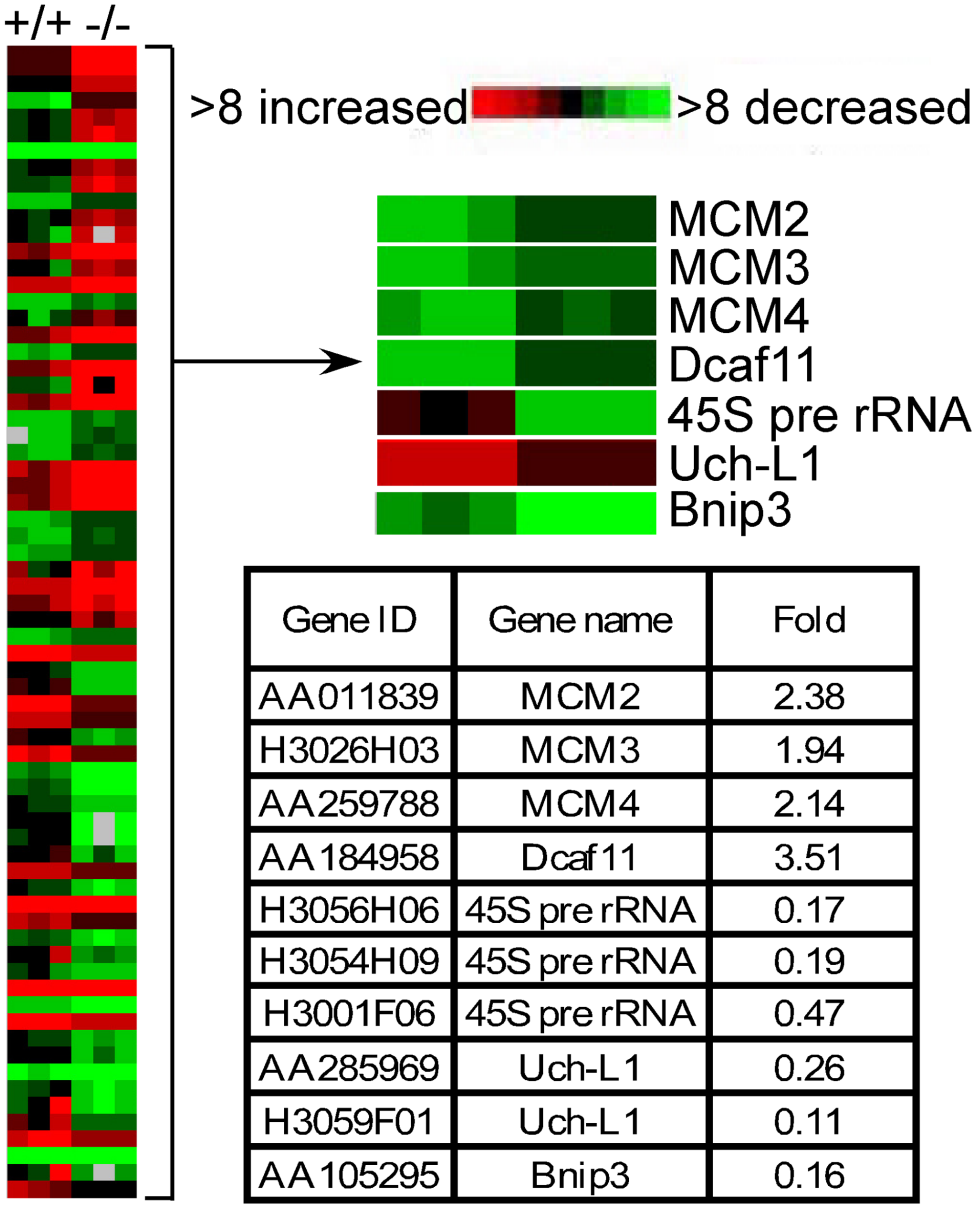
Heat map of significantly changed gene expression from cDNA micro array analysis of wild-type (+/+) and UT-B null (−/−) bladder urothelium. Table below shows mRNA fold change of the significant genes.

To determine the altered gene expression in UT-B null urothelium, a microarray assay was performed. In UT-B null urothelium, 69 genes were significantly altered at mRNA level, of which DDB1 and CUL4 associated factor 11 (Dcaf11) and minichromosome maintenance 2–4 (MCM2-4) were significantly up-regulated, and ubiquitin carboxy-terminal hydrolase L1 (Uch-L1), adenovirus E1B-19K/Bcl-2 interacting protein 3 (Bnip3) and 45 S pre rRNA were down-regulated (Fig. 2). These latter genes are related to DNA damage and apoptosis.

### Ultrastructure of UT-B Null Bladder Urothelium

**Figure 3:**
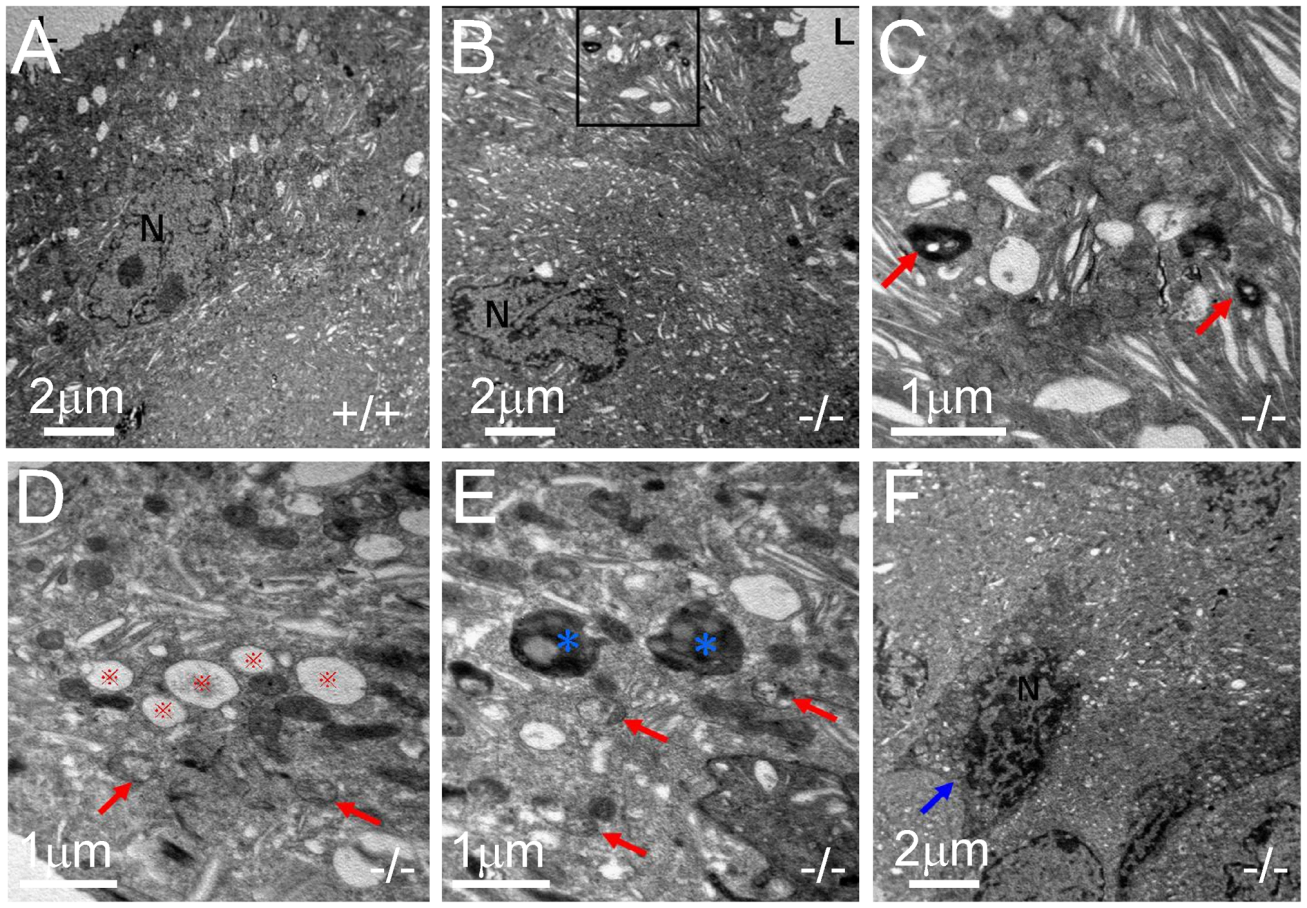
Subcellular structures of urothelial cells in wild-type (A) and UT-B null bladder (B to F) observed by transmission electron microscopy. **A.** Wild-type (+/+) superficial urothelial cell. **B.** The presence of myelin figure formation (in black box) in cytoplasm of UT-B null (−/−) urothelial cell. **C.** High magnification of image in black box in Fig. 3B showing discoidal or fusiform-shaped vesicles and heavily stained myelin figure (arrows) in UT-B null urothelial cell. **D.** Swollen mitochondria (arrows) in cytoplasm of UT-B null urothelial cell. **E.** Swollen mitochondria (arrows) and lysosomes (asterisk) in cytoplasm of UT-B null urothelial cell. **F.** Moderate cell shrinkage, densified cytoplasm and nucleus (N) condensation in cytoplasm of UT-B null urothelial cell.

Light microscopic analysis showed that tissue architecture of the bladder urothelium appeared grossly normal in UT-B null bladder (data not shown). Transmission electron microscope (TEM) analysis was performed to detect the ultrastructure of urothelial cells. At low-magnification, the superficial urothelial cells in wild-type mice appeared normal with discoidal or fusiform vesicles filled in cytoplasmic compartments (Fig. 3A). Most urothelial cells in UT-B null bladder showed normal nuclei with abundant cytoplasm except for some non-specific morphologic changes (Fig. 3B). At high-magnification, we observed an increased myelinfigure formation (Fig. 3C), mitochondrial swelling (Fig. 3D and E), and lysosome formation (Fig. 3E), which indicates non-specific injuries resulting in enhanced cell metabolism and membrane structure degradation. Scattered shrunken urothelial cells with abnormally increased cytoplasm electron density and chromatin condensation were observed in UT-B null bladder urothelium, which suggests an early stage of apoptosis (Fig. 3F).

### Increased Apoptosis in UT-B Null Mouse Urothelium

**Figure 4:**
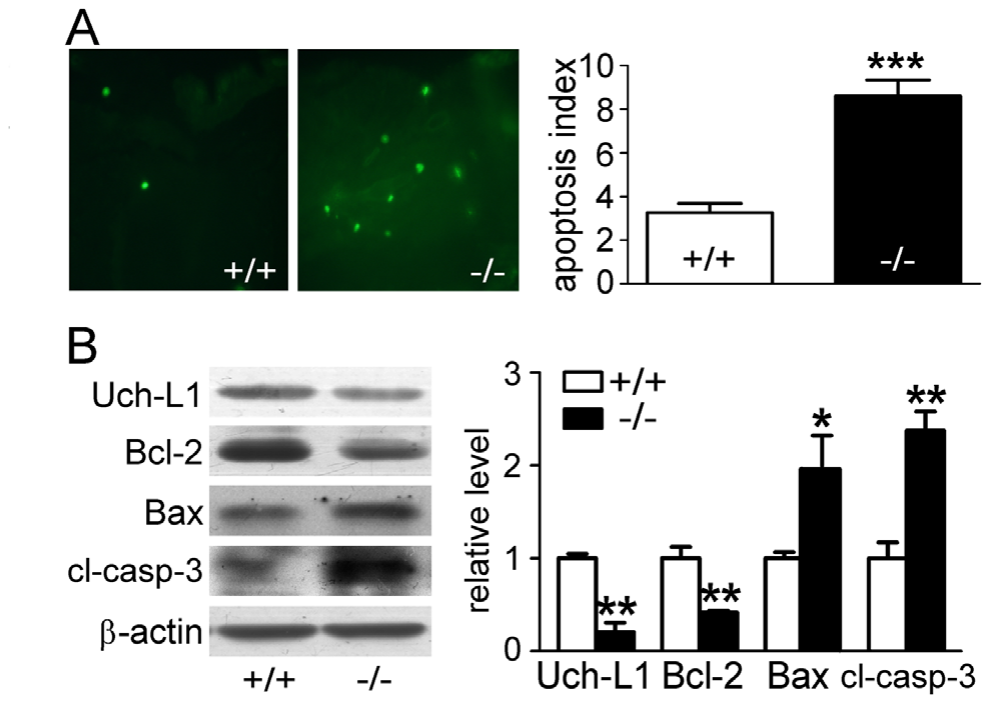
Apoptosis in wild-type and UT-B null bladder urothelium. **A.** Left graph shows the representative pictures of TUNEL in wild-type (+/+) and UT-B null (−/−) bladder urothelium. Bar graph (right) shows the statistic results of TUNEL, average of 5 40X visual fields each sample, Mean ± SEM, ***p<0.001, compared with wild-type mice, n = 6. **B.** Western blot analysis of Uch-L1, Bcl-2, Bax and caspase-3 protein expression. Left graph shows the representative blots. Bar graph (right) shows the density ratios of Uch-L1, Bcl-2, Bax and caspase-3 to β-actin. Mean ± SEM, *p<0.05, **p<0.01, compared with wild-type, n = 4.

A TUNEL Staining Assay was performed to evaluate apoptosis in bladder urothelium. UT-B null urothelium showed more TUNEL positive cells than wild-type (8.6±0.7 vs 3.3±0.4 positive cells/visual field, p<0.001) (Fig. 4A). Western blot analysis of apoptosis-related proteins demonstrated less Bcl-2, more Bax and cleaved caspase-3 expression in UT-B null bladder urothelium. Uch-L1, which has been reported to encourage cell proliferation and boost signaling pathways against apoptosis [13], [14], was seen here to be down-regulated in UT-B null bladder urothelium. These data indicate that more apoptosis occurred in UT-B null urothelium than in wild-type (Fig. 4B).

### Increased DNA Damage in UT-B Null Bladder Urothelium

**Figure 5:**
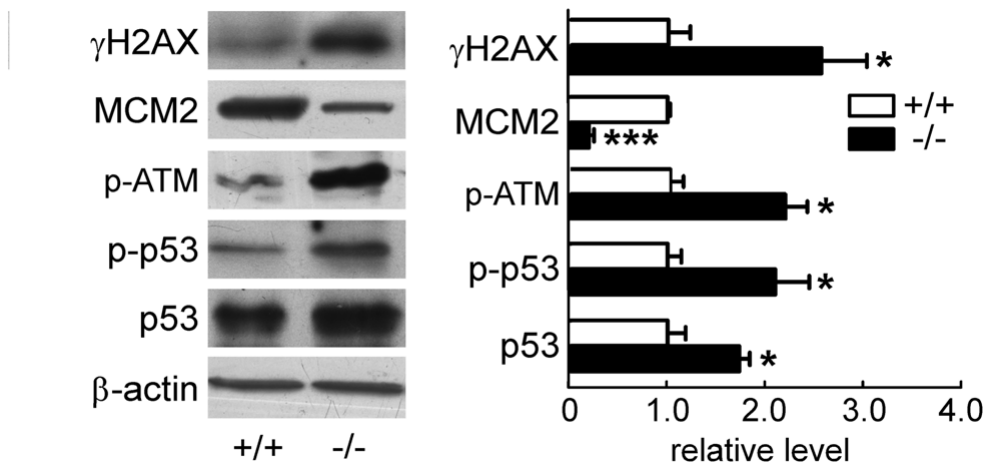
DNA damage in wild-type and UT-B null bladder urothelium. Western blot analyses of proteins related to DNA damage were performed on wild-type (+/+) and UT-B null (−/−) bladder urothelium (left). Bar graph (right) shows the density ratios of γ-H2AX, MCM2, p-ATM, p-p53 and p53 to β-actin. Mean ± SEM, *p<0.05, ***p<0.001, compared with wild-type, n = 4.

To test whether UT-B deletion affects DNA damage, we analyzed phosphorylation of histone H2AX (γH2AX), one of the earliest indicators of DNA damage, and MCM2, an important component of MCM2-7 complex that functions at the DNA replication fork in response to single/double stranded break after DNA damage. Up-regulation of γH2AX and down-regulation of MCM2 expression were confirmed with Western blot. Moreover, phosphorylation of ataxia telangiectasia mutated (ATM) kinase at Ser1981 and both p53 expression and phosphorylation at Ser15 were significantly elevated, which represents DNA damage response (Fig. 5).

### Un-activated Autophagy and ERK Signaling in UT-B Null Bladder Urothelium

**Figure 6:**
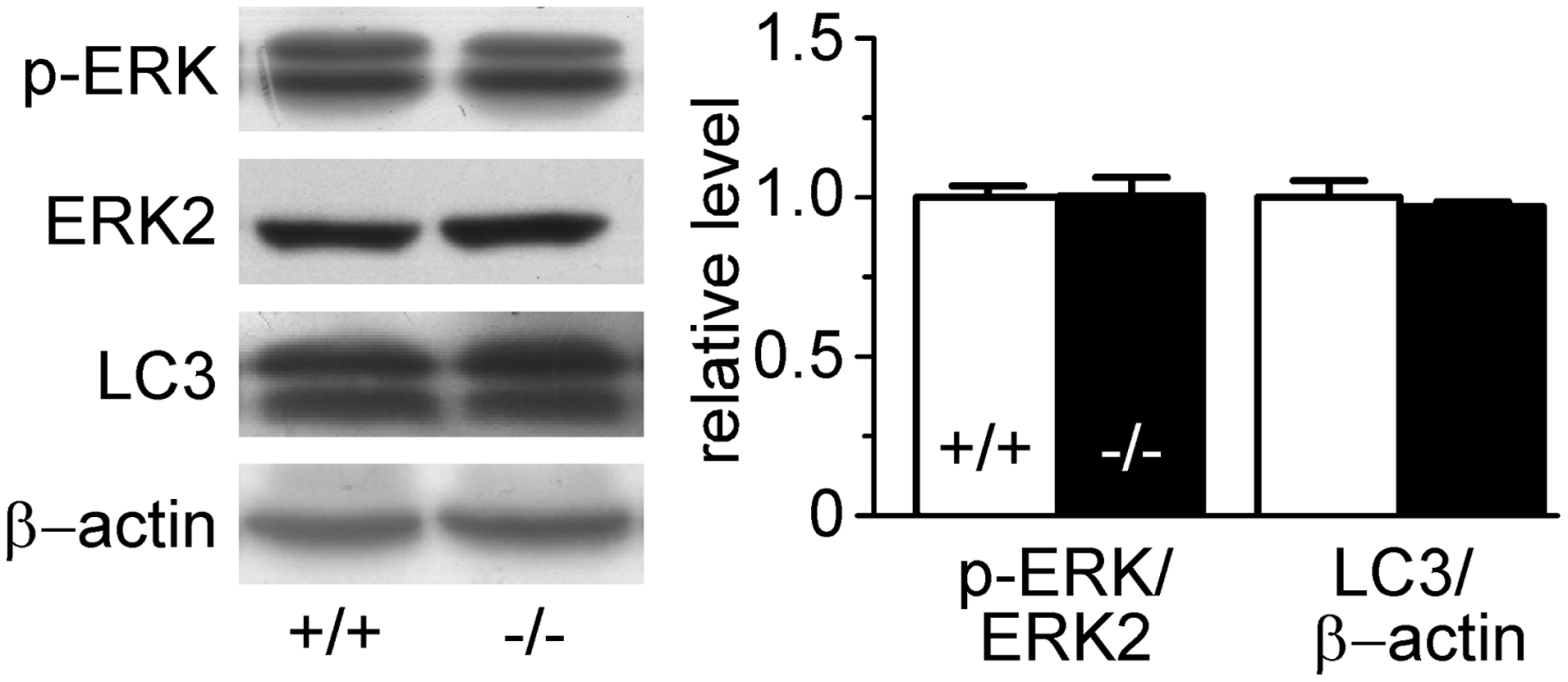
Proliferation and autophagy in wild-type (+/+) and UT-B null (−/−) bladder urothelium. Left graph shows the representative Western blot. Bar graph (right) shows the density ratios of p-ERK and ERK and LC3 to β-actin. Mean ± SEM, n = 4.

To compensate physiological challenges, DNA replication and cell autophagy normally occurs after DNA damage and apoptosis. Hence, we decided to examine the pathways related to these processes. ERK protein expression and phosphorylation levels were the same in UT-B null urothelium as those in wild-type. Western blot showed no conversion from LC3A to LC3B, which is the characteristic of autophagosome (Fig. 6). These results indicate that DNA damage and apoptosis caused by UT-B deletion are not related to ERK signaling and autophagy in bladder urothelial cells.

### Urea Accumulation and Alteration of L-arginine Metabolism Pathway

**Figure 7:**
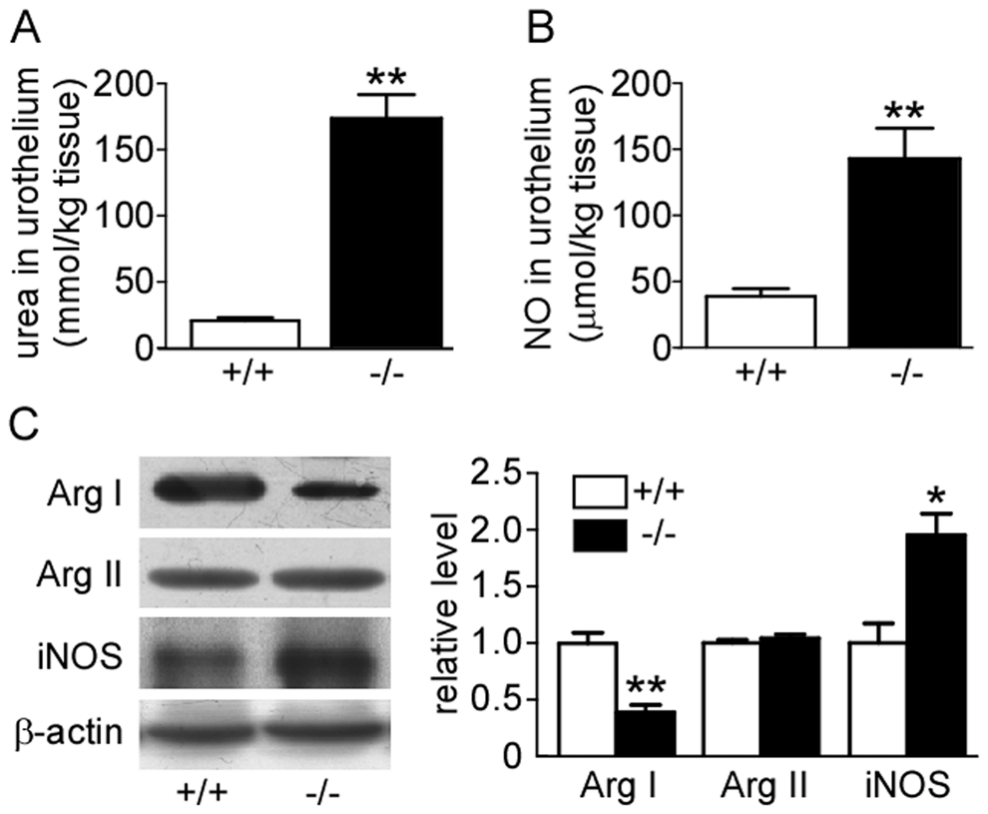
Alteration of L-arginine metabolism system in wild-type (+/+) and UT-B null (−/−) bladder urothelium. **A.** Urea concentrations in bladder urothelium, Mean ± SEM, **p<0.01, compared with wild-type, n = 9. **B.** NO concentration in bladder urothelium, Mean ± SEM, **p<0.01, compared with wild-type, n = 5. **C.** Western blot analysis of arginase I, arginase II and iNOS protein expression. Left graph shows the representative blots. Bar graph (right) shows the density ratios of arginase I, arginase II and iNOS to β-actin. Mean ± SEM, *p<0.05, **p<0.01, compared with wild-type, n = 4.

To explore the mechanism in which UT-B deletion caused apoptosis and DNA damage, urea concentration in bladder urothelium was measured. Urea concentration was almost nine times higher in UT-B null bladder urothelium than in wild-type (176.6±18.2 vs 20.0±1.8 mmol/kg tissue, p<0.01) (Fig. 7A). Because density of blological tissues is very close to 1, these values represent a close approximation of urea concentration in mmol/l.

NO level was also markedly elevated in UT-B null bladder urothelium compared to wild-type (147.4±37.3 vs 41.9±14.6 µmol/kg tissue, p<0.01) (Fig. 7B). Western blot analysis showed that arginase I expression was remarkably decreased in UT-B null urothelium (p<0.01) whereas arginase II expression was unchanged (Fig. 7C). iNOS protein expression was dramatically increased in UT-B null urothelium, while the expression of eNOS and nNOS was barely detectable in urothelium of both genotypes (data not shown). These results suggest that high urea concentration in UT-B null bladder urothelium may lead to unbalanced arginine metabolism. More NO is produced via increased iNOS, while the conversion of arginine to ornithine and urea is reduced, due to repression of arginase I expression.

### Urea Loading Triggered Cell Cycle Delay and Apoptosis

**Figure 8:**
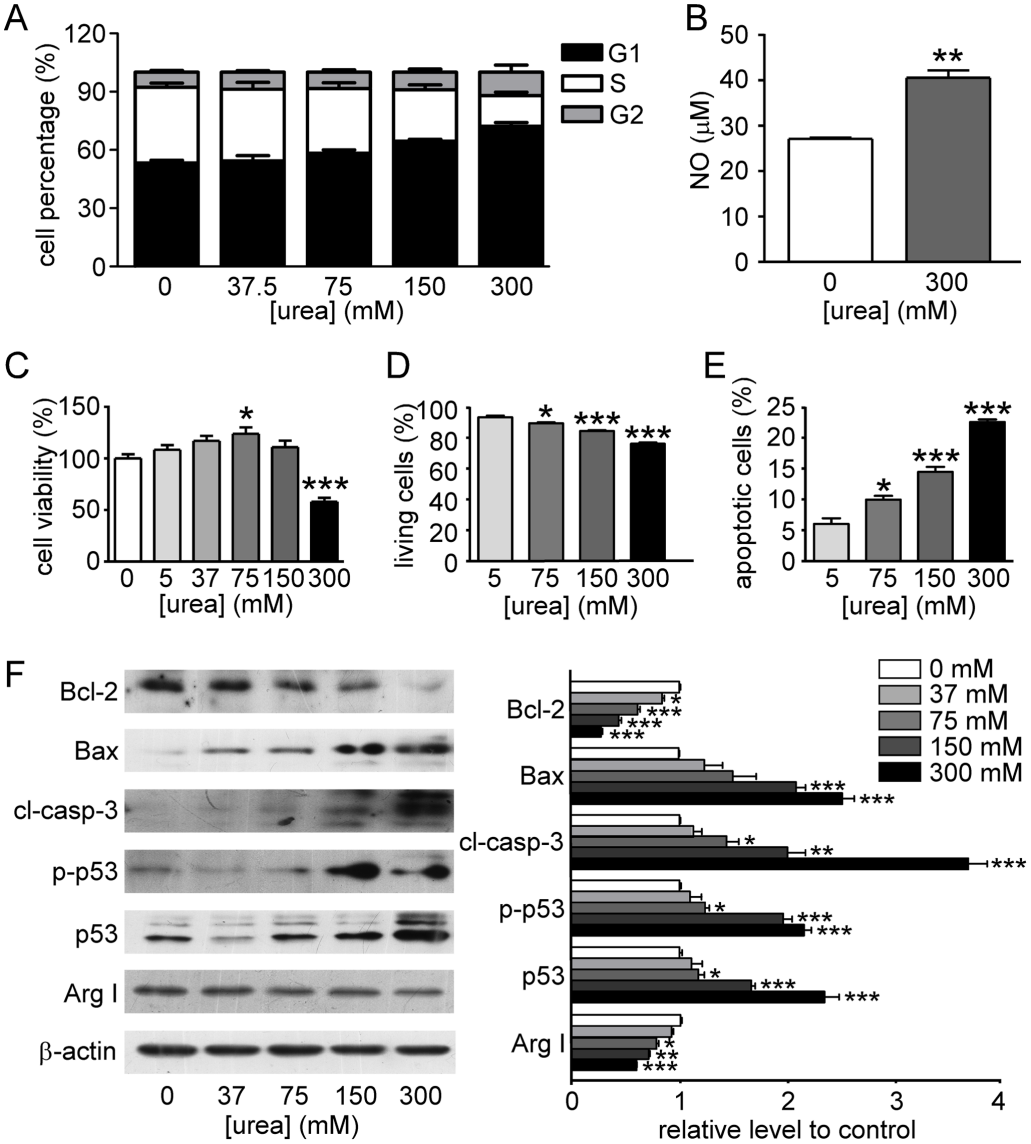
DNA damage and apoptosis detected in T24 cells after incubation with urea. **A.** Effect of urea on cell cycle progression of T24 cells. **B.** NO concentration in supernatant of T24 cell culture. **C.** T24 cell viability detected by CCK-8. **D.** Living cells analyzed by flow cytometry (AnnexinV and PI staining). **E.** Apoptotic cells analyzed by flow cytometry (AnnexinV and PI staining). **F.** Bcl-2, Bax, caspase-3, p-p53, p53, arginase I, and β-actin expression determined by Western blot analysis. Left graph shows the representative blots. Bar graph (right) shows the density ratios of Bcl-2, Bax, caspase-3, p-p53, p53 and arginase I to β-actin. Mean ± SEM, *p<0.05, **p<0.01, ***p<0.001, compared with control, n = 4.

To confirm the effect of urea on DNA damage and apoptosis *in vitro*, T24 cells were cultured in media containing urea at the following concentrations: 5, 37.5, 75, 150 or 300 mmol/l. As shown in Fig. 8A, high urea concentration caused G1 arrest and significantly increased NO concentration (40.6±2.1 vs 27.1±0.4 µmol/L, p<0.01) in the culture medium (Fig. 8B) when cells exposed to 300 mmol/l urea for 3 hours. Cell viability slightly increased with increasing urea concentration but was repressed at 300 mmol/l urea. Cell viability was significantly improved at around 75 mmol/l urea, which was consistent with previously reported data in other cell types [15]. Cell apoptosis was detected with a flow cytometer after staining with annexin-V-FITC and PI. Urea induced apoptosis in T24 cells as demonstrated by the decreased number of annexin V−/PI– cells (live cells) and the increased number of annexin V+/PI+ cells (late apoptotic cells). Urea significantly reduced cell apoptosis in a dose-dependent manner (Fig. 8D and E). Western blot showed an up-regulation of Bax, cleaved-caspase-3, p53 and p-p53, and a down-regulation of arginase I and Bcl-2 in T24 cells incubated in high urea concentration for 24 hours. The results confirm the alterations found in *in vivo* experiments (Fig. 8F).

## Discussion

The motivation for this study was based on the high level of expression of UT-B in the bladder urothelium in both humans and laboratory animals. We speculated that UT-B deletion might cause urea accumulation and abnormal metabolism in bladder urothelial cells, which might result in cell damage and abnormal phenotype in bladder urothelium.

The results confirmed our hypotheses. Urea concentration in bladder tissue was nine-fold higher in UT-B null mice than in wildtype mice. Arginase expression in bladder tissue was reduced while eNos expression was increased, suggesting that accumulation of urea induced a shift from the polyamine pathway to the NO pathway. A number of cell functions were perturbated, possibly because of these metabolic disorders.

The microarray analysis showed that UT-B deletion caused alteration of MCM 2–4, Dcaf11, 45S pre rRNA, Uch-L1 and Bnip3. MCM proteins are required for DNA replication and are a target of S-phase checkpoints [16]. The loss of MCM functionality causes DNA damage and genome instability [17]. MCM expression was up-regulated in proliferating cells, providing a diagnostic marker for both cancerous cells and cells with the potential to become malignant [18], [19]. MCM2-deficient mice show enhanced DNA damage in agreement with a chronic elevation in p53-mediated DDR (DNA damage response) pathway. Moreover, abrogating p53-mediated DDR in MCM2-deficient mice resulted in increased embryonic lethality and accelerated cancer formation [20]. Our data shows a decrease of MCM2 expression in UT-B null bladder urothelium–indicating elevated DNA damage. DNA damage in UT-B null bladder urothelium was confirmed by H2AX and p53 up-regulation and p53 phosphorylation.

As a downstream protein of ATM and a transcriptional inducer of PUMA, NOXA and Bax, p53 is a linkage between DNA damage and apoptosis [21], [22], p53 plays a vital role in initiating the cell cycle delay after exposure to high salt and urea, thus possibly providing sufficient time for repairing DNA damage or for initiating apoptosis, which could prevent the propagation of damaged DNA [23]. The Bcl-2 family members are key players in the mitochondria-dependent intrinsic pathway of apoptosis. Bax is a member of the Bcl-2 family proteins, which can promote apoptosis by forming oligomers on the outer mitochondrial membrane and forming a channel for the release of cyto-c. We found increased phosphorylated ATM, Bax and active caspase-3 and decreased Bcl-2 expression in UT-B null bladder epithelium. This phenomenon demonstrates that functional loss of UT-B in bladder urothelium leads to DNA damage and apoptosis by an ATM-p53-dependent pathway.

Ultra microstructure analysis and TUNEL assay demonstrated that enhanced apoptosis occurs in UT-B null bladder urothelial cells. We found ultrastructural changes such as early stage apoptosis and non-specific morphologic changes including mitochondrial swelling, myelinfigure formation in UT-B null bladder epithelium. Uch-L1 belongs to deubiquitinating enzymes (DUBs), capable of removing ubiquitin from protein substrates. Uch-L1 has been reported to be involved in regulation of microtubule dynamics and attributed to cell mitosis [13]. Recently Uch-L1 was proposed as a marker for a potential involvement in carcinogenesis. Uch-L1 changes cell morphology by regulating cell adhesion through Akt-mediated pathwayby down-regulating the antagonistic phosphatase PHLPP1. This event requires de-ubiquitinase activity [14], [24]. Our data showed a low expression of Uch-L1 in UT-B null bladder epithelium and supports that inhibition of Uch-L1 induced cell cycle arrest and apoptosis [24], [25], [26].

There are multiple factors that could independently or together account for DNA damage and apoptosis. It is interesting that functional deletion of a urea-transporter can influence DNA damage and apoptosis. UT-B has been shown to transport urea and urea analogues selectively without carrying ions and sugars [27]. Urea nitrogen concentrations in the rat and dog bladder are about three-fold higher than in serum and other non-renal tissues [8]. In the present study, urea concentration in the bladder of UT-B null mice is approximately 20 times higher than normal urea concentration in plasma (9.11±0.58 mmol/l) compared to only about 2 times higher in wild-type mice [28]. High urea concentration may change multiple biological activities by destroying hydrophobic bonds of protein structure or causing protein carbamylation [29]–[31]. Acute urea loading has been reported to induce cell cycle delay and apoptosis [32]. Urea also induces oxidative stress in several cell types, as evidenced by the appearance of 8-oxoguanine lesions and single-strand breaks in genomic DNA after urea exposure [33].

Urea is a product of arginine metabolism. Arginase catalyses the hydrolysis of L-arginine to L-ornithine and urea, thus playing an essential role in hepatic and intestinal urea formation. Arginase also provides ornithine, the substrate for polyamines synthesis [34], [35]. In cells expressing arginase, this enzyme is a critical regulator of nitric oxide formation by competing with NOS for their common substrate (L-arginine) [36]–[38]. Arginase activity may thus influence numerous physiological and pathological processes in many tissues, including bladder urothelium [39], [40]. There are two distinct mammalian arginases, arginase I and II, which are encoded by different genes and differ in molecular and immunological properties, tissue distribution, subcellular location, and regulation of expression [41]. Arginase I colocalizes with ornithine decarboxylase (ODC) in the cytosol, which may preferentially direct ornithine to the polyamine pathway, whereas arginase II may preferentially direct ornithine to proline and glutamate production, due to the colocalization of arginase II and ornithine aminotransferase (OAT) in mitochondria [42]. Therefore, the expression of the two arginases was analyzed in bladder urothelium. UT-B deficiency was associated with decreased arginase I expression, but no change in arginase II. The down-regulation of arginase I indicates that the formation of the downstream products, ornithine and polyamine, may decrease. Some studies have found that polyamines, particularly spermine, were involved in the regulation of gene expression, the stabilization of chromatin and the prevention of endonuclease-mediated DNA fragmentation and DNA damage [43]–[45].

Nitric oxide (NO), a radical species produced by many types of cells, is known to participate in collateral reactions and lead to DNA damage and cell death in both NO-generating and neighboring cells [46]. The absolute dominant way of NO generation is from L-arginine with NO synthase (NOS) activation. Arginase competes with NOS for the common substrate L-arginine, therefore, regulating and competitively inhibiting each other [35]. In addition, arginase inhibits the production of nitric oxide (NO) via uncoupling of NOS resulting in the generation of NO scavenger, repression of the translation and stability of inducible NOS protein, inhibition of inducible NOS activity via the generation of urea, and by sensitization of NOS to its endogenous inhibitor, asymmetric dimethyl-L-arginine [36], [47], [48]. This shows significant correlation between activity of iNOS and apoptosis in the urinary bladder urothelium. Furthermore, increased iNOS level in UT-B deficient bladder urothelium, which was supported by reduced arginase I expression, results in an increased NO production. NO inhibits cell proliferation and the mechanism seems to be cGMP-independent and due to the inhibition of ornithine decarboxylase (ODC), therefore, down-regulates polyamine production [49], [50].

## Conclusions

The present study suggests that urea transporter UT-B plays an important role in regulating urea concentration in bladder urothelial cells. UT-B deletion caused a marked urea accumulation in urothelium, which may be responsible for DNA damage and apoptosis possibly by interfering with polyamine production and NO formation and triggering ATM-p53 pathway and elevation of p53 activity. This reduces the ability of the cells to prevent DNA damage and apoptosis. This biological process might result in an increased risk of bladder carcinogenesis.
